# Pharmacogenomic Study of SARS-CoV-2 Treatments: Identifying Polymorphisms Associated with Treatment Response in COVID-19 Patients

**DOI:** 10.3390/biomedicines13030553

**Published:** 2025-02-21

**Authors:** Alexandre Serra-Llovich, Natalia Cullell, Olalla Maroñas, María José Herrero, Raquel Cruz, Berta Almoguera, Carmen Ayuso, Rosario López-Rodríguez, Elena Domínguez-Garrido, Rocio Ortiz-Lopez, María Barreda-Sánchez, Marta Corton, David Dalmau, Esther Calbo, Lucía Boix-Palop, Beatriz Dietl, Anna Sangil, Almudena Gil-Rodriguez, Encarna Guillén-Navarro, Esther Mancebo, Saúl Lira-Albarrán, Pablo Minguez, Estela Paz-Artal, Gladys G. Olivera, Sheila Recarey-Rama, Luis Sendra, Enrique G. Zucchet, Miguel López de Heredia, Carlos Flores, José A. Riancho, Augusto Rojas-Martinez, Pablo Lapunzina, Ángel Carracedo, María J. Arranz, SCOURGE COHORT GROUP

**Affiliations:** 1 Fundació Docència i Recerca Mutua Terrassa, 08221 Terrassa, Spain; ddalmau@mutuaterrassa.cat; 2 Hospital Universitario Mutua Terrassa, 08221 Terrassa, Spain; 3 Fundación Pública Galega de Medicina Genómica (FPGMX), Centro Nacional de Genotipado (CEGEN), Servicio Gallego de Salud (SERGAS), 15706 Santiago de Compostela, Spain; 4 Grupo de Farmacogenómica y Descubrimiento de Medicamentos (GenDeM), Instituto de Investigación Sanitaria de Santiago de Compostela (IDIS), 15706 Santiago de Compostela, Spain; almudena.gil@usc.es (A.G.-R.); sheila.recarey.rama@usc.es (S.R.-R.); 5 Centre for Biomedical Network Research on Rare Diseases (CIBERER), Instituto de Salud Carlos III, 28029 Madrid, Spain; 6 IIS La Fe, Plataforma de Farmacogenética, 43026 Valencia, Spain; 7 Departamento de Farmacología, Universidad de Valencia, 46010 Valencia, Spain; 8 Centro Nacional de Genotipado (CEGEN), Universidad de Santiago de Compostela, 15706 Santiago de Compostela, Spain; 9 Instituto de Investigación Sanitaria de Santiago (IDIS), 15706 Santiago de Compostela, Spain; 10 Centro Singular de Investigación en Medicina Molecular y Enfermedades Crónicas (CIMUS), Universidade de Santiago de Compostela, 15782 Santiago de Compostela, Spain; 11 Department of Genetics and Genomics, Instituto de Investigación Sanitaria-Fundación Jiménez Díaz University Hospital-Universidad Autónoma de Madrid (IIS-FJD, UAM), 28040 Madrid, Spain; 12 Unidad Diagnóstico Molecular, Fundación Rioja Salud, 26006 La Rioja, Spain; 13 Tecnologico de Monterrey, Escuela de Medicina y Ciencias de la Salud and Hospital San Jose TecSalud, Monterrey 64718, Mexico; 14 Instituto Murciano de Investigación Biosanitaria (IMIB-Arrixaca), 30120 Murcia, Spain; 15 Departamento de Ciencias de la Salud, Universidad Católica San Antonio de Murcia (UCAM), 30120 Murcia, Spain; 16 Faculty of Medicine and Health Sciences, Universitat Internacional de Catalunya, 08017 Barcelona, Spain; 17 Grupo de Medicina Genómica, CIMUS, Universidad de Santiago de Compostela, 15782 Santiago de Compostela, Spain; 18 Sección Genética Médica-Servicio de Pediatría, Hospital Clínico Universitario Virgen de la Arrixaca, Servicio Murciano de Salud, 30120 Murcia, Spain; 19 Departamento Cirugía, Pediatría, Obstetricia y Ginecología, Facultad de Medicina, Universidad de Murcia (UMU), 30120 Murcia, Spain; 20 Department of Immunology, Hospital Universitario 12 de Octubre, 28041 Madrid, Spain; 21 Transplant Immunology and Immunodeficiencies Group, Instituto de Investigación Sanitaria Hospital 12 de Octubre (imas12), 28041 Madrid, Spain; 22 Laboratorios MICROLAB Vios, Tegucigalpa 11101, Honduras; 23 Department of Immunology, Ophthalmology and ENT, Universidad Complutense de Madrid, 28040 Madrid, Spain; 24 Centro de Investigación Biomédica en Red de Enfermedades Infecciosas (CIBERINFEC), Instituto de Salud Carlos III, 28029 Madrid, Spain; 25 Genomics Division, Instituto Tecnológico y de Energías Renovables, 38600 Santa Cruz de Tenerife, Spain; cflores@ull.edu.es; 26 Research Unit, Hospital Universitario Nuestra Señora de Candelaria, Instituto de Investigación Sanitaria de Canarias, 38010 Santa Cruz de Tenerife, Spain; 27 Centre for Biomedical Network Research on Respiratory Diseases (CIBERES), Instituto de Salud Carlos III, 28029 Madrid, Spain; 28 Facultad de Ciencias de la Salud, Universidad Fernando Pessoa Canarias, 35450 Las Palmas de Gran Canaria, Spain; 29 Servicio de Medicina Interna, Hospital U.M. Valdecilla, Universidad de Cantabria, IDIVAL, 39008 Santander, Spain; 30 Grupo de Genética, Instituto de Investigación Sanitaria de Santiago (IDIS), 15706 Santiago de Compostela, Spain; 31 Centro de Investigación Biomédica en Red de Enfermedades Raras, Instituto de Salud Carlos III, 28029 Madrid, Spain

**Keywords:** SARS-CoV-2, pharmacogenetics, corticoids, immunomodulators, precision medicine

## Abstract

**Background/Objectives**: The COVID-19 pandemic resulted in 675 million cases and 6.9 million deaths by 2022. Despite substantial declines in case fatalities following widespread vaccination campaigns, the threat of future coronavirus outbreaks remains a concern. Current treatments for COVID-19 have been repurposed from existing therapies for other infectious and non-infectious diseases. Emerging evidence suggests a role for genetic factors in both susceptibility to SARS-CoV-2 infection and response to treatment. However, comprehensive studies correlating clinical outcomes with genetic variants are lacking. The main aim of our study is the identification of host genetic biomarkers that predict the clinical outcome of COVID-19 pharmacological treatments. **Methods**: In this study, we present findings from GWAS and candidate gene and pathway enrichment analyses leveraging diverse patient samples from the Spanish Coalition to Unlock Research of Host Genetics on COVID-19 (SCOURGE), representing patients treated with immunomodulators (*n* = 849), corticoids (*n* = 2202), and the combined cohort of both treatments (*n* = 2487) who developed different outcomes. We assessed various phenotypes as indicators of treatment response, including survival at 90 days, admission to the intensive care unit (ICU), radiological affectation, and type of ventilation. **Results**: We identified significant polymorphisms in 16 genes from the GWAS and candidate gene studies (TLR1, TLR6, TLR10, CYP2C19, ACE2, UGT1A1, IL-1α, ZMAT3, TLR4, MIR924HG, IFNG-AS1, ABCG1, RBFOX1, ABCB11, TLR5, and ANK3) that may modulate the response to corticoid and immunomodulator therapies in COVID-19 patients. Enrichment analyses revealed overrepresentation of genes involved in the innate immune system, drug ADME, viral infection, and the programmed cell death pathways associated with the response phenotypes. **Conclusions**: Our study provides an initial framework for understanding the genetic determinants of treatment response in COVID-19 patients, offering insights that could inform precision medicine approaches for future epidemics.

## 1. Introduction

Coronaviruses are a subfamily of RNA virus that cause a variety of respiratory diseases, including common cold and severe acute respiratory syndrome (SARS). The Beta (β) class of coronaviruses that comprises SARS-CoV-1, MERS-CoV, and SARS-CoV-2 infect the lower respiratory tract, causing a wide variety of symptoms, including cough, anosmia, fever, and headache, among others [1], that can degenerate into pneumonia and may affect the function of other organs, including the liver, heart, kidney, and brain [2]. SARS-CoV-2, responsible for the COVID-19 pandemic, caused 777 million reported cases and 7 million confirmed deaths worldwide, according to recent WHO data. The number of COVID-19 cases and deaths per year have decreased drastically due to vaccination and immunisation of the population. However, newer COVID-19 variants have significantly increased infections, and future coronavirus epidemics are highly likely [3,4]. The high mutation rates of coronaviruses; environmental factors (e.g., temperature, humidity, radiation, and pollution); and, particularly, host susceptibility to infection (e.g., genetic predisposition) may contribute to infection susceptibility [5,6].

To date, there is no specific treatment for the immune reaction produced by SARS-CoV-2 infections [2]. The rapid mutation of coronaviruses may lead to the inefficacy of treatments in the long term, especially those targeting replicative mechanisms [7]. The current COVID-19 treatment options are based on therapies used for previous coronavirus outbreaks (i.e., SARS-CoV-1 and MERS) and include anti-inflammatory compounds such as glucocorticoids, antivirals, antibiotic, antiparasitic, and/or anti-inflammatory compounds previously used for other infectious and non-infectious diseases [8]. About 10–20% of patients do not respond to treatment and develop an aberrant immune system response that results in a cytokine storm, causing severe symptomatology and even death [9]. Monoclonal antibodies such as the immunomodulator Tocilizumab are used for the treatment of severely affected COVID-19 patients suffering from cytokine storms, with varied success [10,11]. Furthermore, one of the leading causes of mortality is the adverse reactions (ADRs) induced by COVID-19 treatments. Tocilizumab and corticoids may cause immunosuppression and increased risk of infection when administered at high doses [12]. A recent study showed a superior incidence (4.75-fold) of ADRs in COVID-19 patients compared to non-COVID patients, with Tocilizumab associated with the higher rate of ADRs [13]. The reasons behind treatment failure and adverse reactions are unclear. Identifying the risk factors for treatment failure may help to better select treatment and/or doses in future epidemics.

Pharmacogenetic studies have revealed genetic variants that may influence the clinical outcome by altering drug metabolic rates and/or drug targets. Genetic variants in hepatic cytochrome P450 (CYP) metabolic enzymes, transporter proteins, targeted cytokines, and virus entry proteins may play an important role in the host response to SARS-CoV-2 infections. For instance, about 85% of the current medications are metabolised by CYP enzymes, which are known to harbour genetic variants affecting their metabolic rates. CYPs functional variants have been reported to influence the response to treatments such as Lopinavir, Ritonavir, and Hydroxychloroquine in several studies [14,15]. CYPs variants may also play an important role in the bioavailability of most corticoids, currently used as the first line of treatment for SARS-CoV-2 infections. The enzymes CYP3A4 and CYP2D6 are the main metabolic pathways of several corticoid compounds, including Dexamethasone, Prednisone, Hydrocortisone, and Fludrocortisone [16]. However, the influence of known CYP functional variants on the response to corticoid treatments has been determined mainly in asthma but not in COVID-19 patients [17]. Solute carrier organic anion transporter family member 1B1 (SLCO1B1), ATP-binding cassette subfamily C member 1 (ABCB1), and ATP-binding cassette subfamily B members 1 and 2 (ABCC1 and ABCC2) transporter proteins also play an important role in the pharmacokinetics of corticoid treatments. Genetic polymorphisms in *SLCO1B1*, *ABCB1*, *ABCC1*, and *ABCC2* have been associated with the response to the antiretroviral Lopinavir and antibiotics like Azithromycin [18,19]. However, the influence of these genetic variants on the response to treatment of COVID-19 infections has not been investigated to date. Proteins linked to host susceptibility to infections may also be related to the treatment response and sustained inflammation [20]. In our own studies, we have shown that variants in genes encoding diverse immunoregulatory interleukins (IL4, IL6, and IL10) are associated with susceptibility to invasive pneumococcal disease [21]. Genetic variants in *IL6*, *IL10*, and C-reactive protein (CRP) genes have also been associated with the severity and community-acquired pneumonia [22]. Immunomodulator treatments directed to inhibit the cytokine storm and, specifically, the *IL6* pathway in severe patients may be affected by these genetic polymorphisms. Several studies have described associations between genetic polymorphisms within the IL-6 receptor (IL6R) gene and Tocilizumab response [23]. Understanding the host pharmacogenetic profile can provide useful information to help fight the virus infection and reduce mortality. However, further evidence is required before using pharmacogenetic information for the personalisation of COVID-19 treatments.

In summary, there are currently few predictors of the clinical response to COVID-19 pharmacological treatments. The main aim of our study is the identification of host genetic biomarkers that predict the clinical outcomes of COVID-19 pharmacological treatments. This information will help to personalise the treatment of COVID-19 and other coronavirus infections, improve its efficacy, and reduce patient morbidity and mortality.

## 2. Materials and Methods

### 2.1. Patients and Sample Selection

Samples from patients diagnosed with COVID-19 were sourced from the Spanish Consortium for COVID-19 Research (SCOURGE; https://github.com/CIBERER/Scourge-COVID19, accessed on 1 April 2022). The samples were obtained between March and December 2020 in 34 medical centres across 25 Spanish cities. A previous publication contains a detailed description of the study cohort [6]. Briefly, COVID-19 infection was diagnosed through PCR testing or clinical assessment. Sample collection and data management were carried out by biobanks associated with the participating centres following informed consent. The whole project was approved by the Galician Ethical Committee (ref. 2020/197) on 10 April 2020. Additional approval was obtained by Ethics and Scientific Committees of the participating centres. All samples and data were processed using standardised procedures, with data management facilitated through REDCap electronic data capture tools hosted at the Centro de Investigación Biomédica en Red (CIBER). For this study, we selected exclusively the patients treated with corticoids and immunomodulators that are known to modulate the cytokine storm, the critical factor that leads to deterioration and mortality in COVID-19 disease.

#### 2.1.1. Clinical Sample

Patients who had received corticoid (*n* = 2202; 1442 males and 760 females, mean age = 66 years, SD = ±15) or immunomodulator treatments (*n* = 849; 633 males and 216 females, mean age = 63 years, SD = ±12) were included in the study. The combined sample of patients treated with immunomodulators and/or corticoids group consisted of *n* = 2487 (1651 males and 836 females, mean age = 66 years, SD = ±14) (see Table 1).

#### 2.1.2. Response Phenotype

Treatment response was assessed using the following data available in all participating centres: survival at 90 days, admission to the intensive care unit (ICU), radiological affectation, and type of ventilation. More detailed or specific response information (i.e., levels of ferritin; interleukins such as IL-6, IL-1β, and IL-10; C-reactive protein (CRP); D-dimer; lactate dehydrogenase (LDH); Troponin; or Prothrombin time (PT)) was not available for most participants.

Survival at 90 days (yes/no) refers to whether the patients survived up to 90 days after hospital discharge. Patients who did not survive at 90 days included patients who passed away in hospital and patients who were discharged but the sequelae of the coronavirus were too severe and passed away soon after. Patients who died more than 90 days after discharge were considered to have died due to causes other than the coronavirus infection. Admission to the intensive care unit (ICU) (yes/no) was commonly used for severe patients with chances of survival during what is known as the first wave of COVID-19 and may have been influenced by the age and severity of the patients. The radiological affectation (yes/no) refers to the changes or abnormalities that can be observed in radiographic images (X-ray or CT scan findings) in the lungs of patients infected with SARS-CoV-2. These changes may include opacities (areas whiter than normal, indicating fluid accumulation or inflammation in lung tissues); infiltrates (presence of fluid in the lung spaces, which may indicate inflammation or damage); and consolidations (accumulation of fluid, inflammatory cells, or scar tissue) that are indicative of the presence and severity of COVID-19 infection in the lung tissue. In our study, we established the radiological affectation as a binary variable to distinguish between patients who received treatment that severely reduced the inflammation and did not develop radiological affectation and patients that did not improve and developed lung affectation. Radiological affectation values have been employed in predicting disease states, particularly amidst the unprecedented circumstances of the pandemic. While the specific dates of the radiological assessments were not documented, it is presumed that patients underwent treatment upon admission to the hospital, followed by subsequent imaging evaluations [24]. The type of ventilation is a categorical variable (1 = no ventilation; 2 = conventional oxygen therapy; 3 = nasal cannulas and IMV) directly related to the state of the disease. If patients fail to respond adequately to medication, disease progression ensues, resulting in an increased requirement for oxygen therapy. As the disease advances, oxygen therapy may need to be escalated to more invasive measures to prevent hypoxia, a severe and potentially fatal condition characterised by viral pneumonia and marked decreases in blood oxygen levels. This progression can lead to acute respiratory distress syndrome (ARDS), organ failure, and, ultimately, death.

Most patients survived more than 90 days after being discharged, with an overall survival rate of 83.8%. Interestingly, the survival rate was slightly higher among patients treated with immunomodulators (85.8%) compared to those treated with corticoids (82.5%). Among patients receiving immunomodulators, approximately half required admission to the ICU, whereas only 23% of patients receiving corticoids were admitted. Additionally, half of the patients in the immunomodulator cohort required invasive ventilation, while 50% of patients in the corticoid and combination therapy cohorts required non-invasive ventilation. Regarding the radiological findings, only 5% of patients in the corticoid and combination therapy cohorts showed no radiological abnormalities, whereas, in the IMM cohort, only 1.37% of patients were unaffected radiologically.

### 2.2. SNP Genotyping

The samples were genotyped using the Axiom Spain Biobank Array (Thermo Fisher Scientific, Waltham, MA, USA) in accordance with the manufacturer’s instructions at the Santiago de Compostela Node of the National Genotyping Centre (CeGen-ISCIII; http://archivo.xenomica.org, accessed on 1 April 2022). This array interrogates 757,836 polymorphisms, including rare variants from exonic regions specifically chosen from the Spanish population’s genetic profile. Quality control (QC) of the GWAS results was performed by the Santiago de Compostela Node of the National Genotyping Centre, as previously described [6]. Briefly, the QC was carried out using the PLINK1.9 package and the R platform 4.3.1. Variant exclusion criteria: Variants with minor allele frequency (MAF) < 1%, call rate < 98%, and Hardy–Weinberg equilibrium (HWE) [*p* < 1 × 10^−10^ as recommended [25]] were excluded from the analyses. X chromosome variants were excluded from the GWAS study and analysed separately in the candidate gene analyses. Sample exclusion criteria: Individuals with a call rate < 98% or whose heterozygosity rate deviated >5 standard deviation (SD) from the mean heterozygosity and individuals with an estimated probability < 20% of pertaining to European ancestry were excluded. In related individuals, one individual of each pair of second-degree relatives were excluded (PI_HAT  >  0.25). After QC, 588.117 variants and 1948 patients who had received corticoids and 696 patients with immunomodulator treatments and with available clinical information were considered. The combined sample of patients treated with immunomodulators and/or corticoids consisted of *n* = 2181.

#### Variant Imputation

Genetic variants were imputed using TOPMed version r2 reference panel (GRCh38 [26]) on the TOPMed Imputation Server (https://imputation.biodatacatalyst.nhlbi.nih.gov/, accessed on 3 October 2023). The following post-imputation filtering criteria were applied for inclusion: coefficient of determination R-square (Rsq > 0.3), HWE *p* > 1 × 10^−6^, and MAF > 1%. This dataset encompassed a total of 15,997,581 genetic markers. Bcftools (version 1.18) a software for managing genetic databases, was used for the SNPID annotation.

### 2.3. Candidate Gene Analysis

We selected polymorphisms within genes implicated in the severity of the disease, metabolism, and targets of compounds used for the treatment of COVID-19 (see Table 2). Specifically, the selected genes were:

- Genes encoding for cytochrome P450 (CYPs) enzymes involved in the metabolism of corticoids: *CYP2C8*, *CYP2C9*, *CYP2C19*, *CYP2D6*, *CYP3A4*, and *CYP3A5* [9,14,18,23,27,28].

- Transporter genes related to the bioavailability of drugs: *ABCB1*, *ABCB11*, *ABCC1*, *ABCC13*, *ABCC2*, *ABCC4*, and *UGT1A1* [9,18,23,27,29,30,31,32].

- Genes directly related to the viral entry into cells of some coronaviruses: *ACE*, *ACE2*, and *ABO* [18,23,29].

- Genes related with the response to immunomodulators and interferon: *FCGR3A*, *IFNAR1*, *IFNG*, *IFNGR1*, *IFNGR2*, *IFNAR2*, *IFNLR1*, *IFNA16*, *IL-1A*, *IL1B*, *IL2*, *IL4*, *IL6*, *IL6R*, *IL10*, *IRF7*, and *TNFA* [9,18,23,27,29,33].

- Genes related with the COVID-19 infection-induced cytokine storm: *TLR10*, *TLR8*, *TLR7*, *TLR5*, *TLR4*, *TLR3*, *TLR2*, *TLR1*, *IL1R1*, *IL-1A*, *IL1B*, *IL2*, *IL4*, *IL6*, *IL6R*, *IL10* & *TNF-α*, *IFNAR1*, *IFNAR2*, *IFITM3*, *IFNG*, *IFNGR1*, *IFNGR2*, *IFNLR1*, *IFNA16*, and *IRF7* [29,33,34,35,36].

### 2.4. Statistical Analyses

The association of genetic variants with the selected phenotypes was investigated using linear and logistic regression models and the Plink v1.9 package [37]. The models were adjusted for covariates known to be associated with the outcome of the disease (age and sex) [6], as well as the first 10 ancestry-specific principal components (PCs). Significance was determined at *p* < 5 × 10^−8^ for the GWAS results. Bonferroni corrections for multiple analyses were applied for the candidate gene results, where the significance threshold was 0.05/number of variants included in the analysis for each gene. All the variants with a *p*-value lower than the threshold established for each gene were considered significant. Analyses were performed separately for each treatment and phenotype.

### 2.5. Pathway Enrichment Analyses

A gene set enrichment analysis was performed using the WEB-based Gene SeT AnaLysis Toolkit (www.webgestalt.org, accessed on 26 February 2024) to extract Gene Ontology terms (including cellular component, biological process, and molecular function ontologies).

## 3. Results

**Figure 1:**
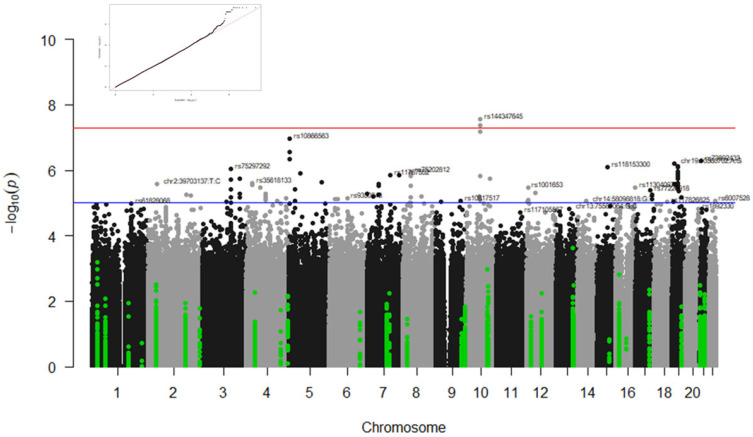
Manhattan plot of results for the type of ventilation in the subgroup of patients treated with immunomodulators. The results of the candidate gene analyses are highlighted in green and the GWAS results in grey and black. The blue line indicates the threshold *p*-value < 1 × 10^−5^ and the red line the threshold *p*-value < 5 × 10^−8^. The corresponding QQ plot is in the top left corner.

**Figure 2:**
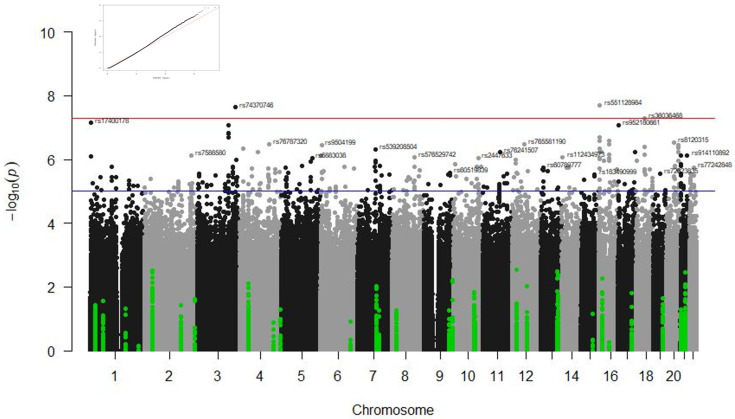
Manhattan plot of the results for radiological affectation in the subgroup of patients treated with corticoids. The results of the candidate gene analyses are highlighted in green and the GWAS results in grey and black. The blue line indicates the threshold *p*-value < 1 × 10^−5^ and the red line the threshold *p*-value < 5 × 10^−8^. The corresponding QQ plot is in the top left corner.

**Figure 3:**
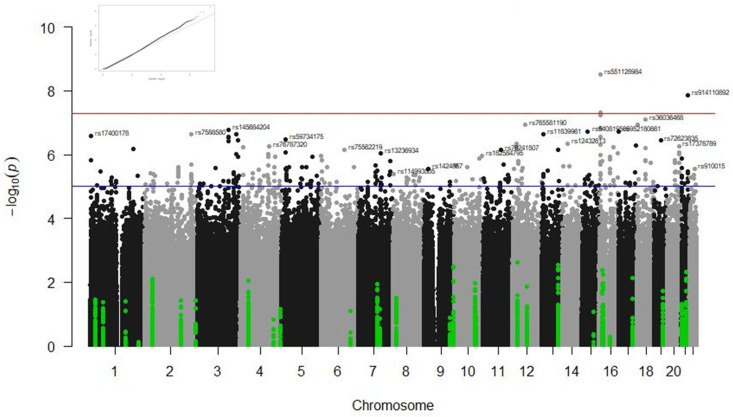
Manhattan plot of the results for radiological affectation in the total combined sample. The results of the candidate gene analyses are highlighted in green and the GWAS results in grey and black. The blue line indicates the threshold *p*-value < 1 × 10^−5^ and the red line the threshold *p*-value < 5 × 10^−8^. The corresponding QQ plot is in the top left corner.

The following sections will describe the results by response phenotypes (survival at 90 days, admission in ICU, type of ventilation, and radiological affectation) and type of study (GWAS or candidate gene studies). Table 3 and Table 4 summarise the results from the GWAS analyses and candidate gene studies, respectively. Figure 1, Figure 2 and Figure 3 illustrate the significant results from these analyses.

### 3.1. GWAS Results

GWAS statistical analyses revealed several genetic loci associated with the type of ventilation in the IMM cohort and the radiological affectation in the COMB and CORT groups at the genome-wide significance level. No statistically significant association was observed when analysing ICU stay and survival at 90 days. Figure 1, Figure 2 and Figure 3 illustrate the Manhattan plots of the most significant results.

#### 3.1.1. Associations with Type of Ventilation

Numerous variants in the gene *ANK3* (rs144347645, rs16915354, rs145299149, *p* rs142740585, rs115701266, rs116165734, rs16915359, rs16915361, rs149847098, and rs144806783) were associated with the type of ventilation in the IMM group (*p* < 5 × 10^−8^ for all comparisons; see Table 3 and Figure 1).

#### 3.1.2. Associations with Radiological Affectation

Significant associations were found between radiological affectation and variants in the gene *RBFOX1* in the CORT (rs551128984, *p* = 2.01 × 10^−8^) and in the COMB (rs551128984, *p* = 3 × 10^−9^ and rs72765129, *p* = 4.75 × 10^−8^) groups. Variants regulating the expression of the gene *ZMAT3* were also significantly associated with this phenotype in the CORT group (rs74370746 and rs78451671, *p* = 2.33 × 10^−8^). A variant in the *MIR924HG* gene was associated with radiological affectation in the CORT group (rs36036468, *p* = 4.99 × 10^−8^). Two variants in the *ABCG1* gene were associated with radiological affectation in the COMB sample (rs914110892 and rs112302620, *p* = 1.38 × 10^−8^) (see Table 3 and Figure 2 and Figure 3).

#### 3.1.3. Associations with ICU and Survival at 90 Days

No statistically significant associations were found at the genome-wide level (*p* < 5 × 10^−8^) for the ICU admission and 90-day survival phenotypes in any of the comparisons performed in the different groups.

### 3.2. Candidate Genes Results

Single marker analyses of selected variants in the candidate genes revealed several significant associations after correcting for multiple analyses (see Table 4).

#### 3.2.1. Associations with Survival at 90 Days

Several genes involved in the immune response (*TLR5*, *IFNG-AS1*, *TLR1*, *TLR6*, and *TLR10*) contained genetic variants associated with survival at 90 days. Associations were found between survival and the *TLR5* variants in the IMM cohort (rs55866312 and rs542741410, *p* < 5.39 × 10^−3^). Associations were also found with the *IFNG-AS1* gene variants in the CORT group (rs12306899 and rs12300716, *p* = 4.05 × 10^−5^) and the combined COMB cohort (rs12300716, rs12306899, rs10878747, rs10878749, rs7301797, rs7306440, rs2870955, rs7133171, rs7137158, and rs11177059, *p* < 4.74 × 10^−5^). Significant associations were observed with variants located in an overlapping region shared by the genes *TLR1* and *TLR6* (rs11933455, rs111530790, rs6849400, rs146468588, rs376523214, rs111980996, rs113668069, and rs148035117, *p* < 3.5 × 10^−4^) in patients treated with corticoids. We identified one significant polymorphism in the *TLR10* gene (rs149895872) associated with 90-day survival in the CORT (*p* = 9.05 × 10^−4^) and in the COMB (*p* = 1.05 × 10^−3^) groups.

#### 3.2.2. Associations with Admission to the Intensive Care Unit (ICU)

Significant associations were found between admission to the ICU and a variant in the *ABCB11* gene in the IMM cohort (rs3770585, *p* = 2.55 × 10^−4^). Associations were also found with variants in the *CYP2C19* (rs12258243 *p* = 3.19 × 10^−4^) and *ACE2* (rs62578917, *p* = 3.26 × 10^−4^) genes.

#### 3.2.3. Associations with the Type of Ventilation

Statistical analyses revealed a significant association with a variant (rs6742078) in the complex region of *UGT1A*, a multigenic region that generates nine *UGT* proteins in the CORT (*p* = 5.12 × 10^−4^) and COMB (*p* = 6.73 × 10^−4^) groups. Two associations were found in variants of the *TLR4* gene (rs12377632 and rs7868859, *p* < 6.35 × 10^−4^) in the CORT patients.

#### 3.2.4. Associations with Radiological Affectation

Associations were found between variants in the *IL1A* gene (rs3783585, rs2071375, and rs697) and radiological affectation in the CORT (*p* = 7.83 × 10^−5^, *p* = 5.63 × 10^−4^, and *p* = 5.63 × 10^−4^, respectively) and COMB (*p* = 6.406 × 10^−5^, *p* = 1.128 × 10^−3^, and *p* = 1.128 × 10^−3^, respectively) groups (Figure 2 and Figure 3).

### 3.3. Functional Enrichment Analyses Results

A gene set enrichment analysis was performed for each cohort using the top 5000 genes from the GWAS results ranked by the lowest unadjusted *p*-values using the Gene Ontology databases (Biological Process, Cellular Component, and Molecular Function).

Biological Process (BP): Overrepresentation of genes involved in the regulation of neuron projection development, regulation of transsynaptic signalling, regulation of membrane potential, and dendrite development was detected in the three cohorts. Other pathways that appeared significantly enriched were small GTPase-mediated signal transduction and developmental growth involved in morphogenesis within the IMM cohort, renal system development, and cell–substrate adhesion in the CORT subgroup and muscle system process and sodium ion transport in the COMB group. Genes involved in the regulation of developmental growth appeared enriched in the IMM and CORT cohorts, while the genes involved in cell junction assembly appeared significantly enriched in the CORT and COMB cohorts.

Cellular Component (CC): Genes involved in nervous system and structural components like adherent junctions were enriched in all the cohorts.

Molecular Function (MF): Most of the enriched molecular functions were related to binding domains, such as alcohol, actin, and steroid binding (IMM); calmodulin and phospholipid binding (CORT); phosphoprotein binding (COMB); and scaffold protein and PDZ domain binding, in more than one cohort. There is also gene enrichment in transporter activity, including organic acid transmembrane, metal ion, monoatomic ion, and gated channel activity, present in all the analysed cohorts. Cyclase and nucleoside-triphosphatase regulator activities were found enriched in various cohorts. Enrichment in adhesion and motor activity was observed in the IMM and CORT cohorts, respectively. Finally, enrichment of glutamate receptor activity was observed in all the cohorts.

## 4. Discussion

Given the severity of SARS-CoV-2 and the unfortunate deaths resulting from inadequate drug responses, identifying predictive factors could tailor treatment in future coronavirus outbreaks [38]. In this study, we investigated genetic predictors of treatment effectiveness in a large group of COVID-19 patients. Several polymorphisms in the genes involved in immune response and previously associated with infection severity were found to be associated with the treatment response, amongst others.

### 4.1. Genes Related to Response to Immunomodulators

Several genetic variants in the genes involved in immune response or coding for transporter proteins were associated with the response to immunomodulators.

**Figure 4:**
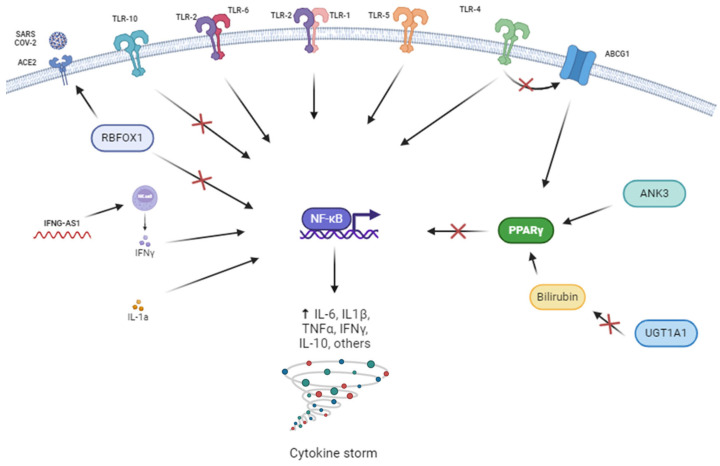
Interactions between the significative results of the GWAS and candidate genes analysis and the pro-inflammatory molecule Nuclear Factor kappa-B. Created with BioRender.com.

GWAS analyses revealed several ankyrin3 (*ANK3*) polymorphisms associated with the type of ventilation in the IMM cohort. *ANK3* is a gene mostly related to neuronal development [39,40,41]. A previous study identified the potential role of *ANK3* in the PPARα/PPARγ signalling pathway and immune infiltration [39] (see Figure 4). Minor alleles of *ANK3* genetic variants were associated with a lower probability of needing invasive ventilation.

Candidate gene association studies revealed several statistically significant associations after Bonferroni corrections. An ATP-binding cassette subfamily B member 11 (*ABCB11*) polymorphism was associated with admission to the ICU. *ABCB11* encodes a protein belonging to the ATP-binding cassette (ABC) transporter superfamily, which facilitates the movement of various molecules across cellular membranes [42]. The *ABCB11* rs3770585-A allele was associated with a higher probability of being admitted to the ICU of patients treated with immunomodulators. Several genetic polymorphisms in the family of Toll-like receptors (TLRs) were associated with survival at 90 days. It is well known the function of TLRs in inflammation [43,44,45,46], and TLRs have been suggested as possible targets for treatments against COVID-19 [47,48]. TLRs regulate cytokine expression and indirectly trigger the adaptive immune system through the secretion of pro-inflammatory cytokines such as IL-1, IL-6, and tumour necrosis factor-alpha (TNF-α) [45] (see Figure 4). Minor alleles of *TLR5* variants were associated with a lower probability of survival in patients treated with immunomodulators and may help to identify patients requiring alternative treatments.

It is important to note that just a limited number of significant associations were observed in response to the immunomodulators, probably due to the moderate sample size of the cohort of patients treated with Tocilizumab or similar molecules. However, the involvement of the genes associated with IMM treatment with the modulation of the immune response and transport suggests that these could be plausible findings. Nevertheless, confirmation of these associations in a larger sample of IMM-treated patients is required.

### 4.2. Genes Related to the Corticoid Response

GWAS and candidate gene studies showed that most of the genes associated with the response to corticoids are involved in inflammation and the immune response.

An RNA-binding fox-1 homolog 1 (*RBFOX1*) polymorphism was associated with radiological affectation in patients treated with corticoids in the GWAS study. This gene regulates tissue-specific alternative RNA splicing. A previous review proposed that overexpression of *RBFOX1* inhibits inflammation and oxidative stress-related factors repressing (NF-κB) [49] (see Figure 4). Another study analysing the influence of *RBFOX1* in SARS-CoV-2 infection suggested that *RBFOX1* may act as an upstream regulator for *ACE2* [50]. In our study, the “C” allele of the rs551128984 variant was associated with a lower probability of developing radiological affectation, contributing evidence to the involvement of this gene in SARS-CoV-2 infection and treatment. Other genetic variants not related to inflammation or the immune response were also observed in the GWAS study to be associated with the corticoid response. Two zinc finger matrin-type 3 (*ZMAT3*) polymorphisms were associated with radiological affectation. This gene is implicated in the regulation of alternative splicing processes, influencing the stability and translation function of RNA [51]. Minor alleles of the rs74370746 and rs78451671 variants were associated with a lower probability of developing radiological affectation. A previous GWAS study comparing symptomatic and asymptomatic patients of COVID-19 suggested a potential correlation between genetic variability in *ZMAT3* and COVID-19 severity in the Chinese population [52], a finding that would endorse a possible role in relation to treatment response, although different risk polymorphisms were identified in both studies. A MIR924 host gene (*MIR924HG*) polymorphism was also associated with radiological affectation in corticoid-treated patients. MIR924HG is a IncRNA that regulates the expression of *CELF4*, a gene involved in alternative mRNA splicing [53]. The “T” allele of the rs36036468 variant was associated with a lower probability of developing radiological affectation. However, the relation between MIR924HG and the response to corticoids and/or SARS-CoV-2 infections is still to be discerned.

Several polymorphisms in the TLRs family of genes were associated with survival at 90 days in the candidate gene analyses. As explained before, TLRs regulate the secretion of pro-inflammatory cytokines [45] (Figure 4). Minor alleles in the *TLR1*, *TLR6*, and *TLR10* genetic variants were associated with a lower probability of survival at 90 days in patients treated with corticoids. The “C” allele in the rs12377632 variant of the *TLR4* gene was associated with a decreased likelihood of requiring more invasive ventilation, whereas the “G” allele of the rs7868859 variant of the same gene was associated with an increased likelihood of needing more invasive ventilation. Those two variants are in linkage disequilibrium, where the major allele on one variant is correlated with the minor allele of the other. Although no previous study has related TLR and response to corticoids, Dexamethasone inhibits important pathways in the host defence against SARS-CoV-2, such as TLR7 and IFIH1/MDA5 [54], exposing TLRs as a possible target for this treatment. Our results suggest that the *TLR1*, *TLR4*, *TLR6*, and *TLR10* variants may influence the outcome during corticoid treatment in COVID-19 patients.

A polymorphism in the cytochrome P450 family 2 subfamily C member 19 (*CYP2C19*) gene was associated with admission to the ICU. *CYP2C19* was selected for its role in the metabolization of many xenobiotics, including anticonvulsive drugs, Clopidogrel, Omeprazole, Diazepam, some barbiturates, and certain corticoids [55]. The *CYP2C19* rs12258243-A allele was associated with a higher probability of corticoid-treated patients being admitted to the ICU. Several studies have shown the influence of corticoids on the expression of *CYP2C19* and other CYPs [56,57]. The rs12258243 variant is in linkage disequilibrium with the rs12248560 variant (r^2^ = 0.84) that predicts *CYP2C19* ultrarapid activity. Our results suggest that the *CYP2C19* rs12258243 variant is associated with corticoid metabolism alterations that contribute to variability in the treatment response. Another gene related to drug availability may influence the corticoid response: An UDP glucuronosyltransferase 1A1 (*UGT1A1*) polymorphism was associated with the type of ventilation. The main function of UGT1A1 is degrading bilirubin, a hormone that activates the PPARα receptor and reduces the inflammation by reducing the production of PCR, TNF-α, and IL-6 [58] (see Figure 4). The “T” allele of the rs6742078 variant was associated with a higher probability of needing invasive ventilation. Previous studies have related UGT1A1 with the response to antivirals [59]. One study has related UGT1A1*6 (rs4148323) with the response to (CDE-11), a treatment for lymphoma that includes Dexamethasone, Irinotecan, and other compounds [60]. Thus, these results contribute further evidence of the relation of *UGT1A1* variants with response to corticoid treatment.

A genetic variant in angiotensin-converting enzyme 2 (ACE2) was associated with admission to the ICU. The *ACE2* rs62578917-A allele was linked to a higher probability of ICU admission of patients treated with corticoids. However, the effect of corticoids on the expression of ACE2 remains unclear. The *ACE2* gene was selected for study due to the role of its encoded protein in facilitating the entry of the SARS-CoV-2 virus in the host cells [61]. Regarding the relationship between ACE2 and the response to corticosteroids, some researchers have observed reduced ACE2 expression in chronic obstructive pulmonary disease [62]. However, another study reported increased ACE2 expression in asthmatic patients using inhaled corticoid therapies [63]. Nevertheless, our findings suggest that the *ACE2* rs62578917 variant may influence the response to corticoid treatments in COVID-19 patients.

Several candidate genes involved in the cytokine storm induced by the infection were associated with the response to corticoid treatments. Three *IL1A* polymorphisms were associated with radiological affectation in patients treated with corticoids. The IL-1 family comprises various pro- and anti-inflammatory proteins, including IL-1α and IL-1β, which exert pro-inflammatory effects by binding to active and inactive receptors. IL-1α-mediated inflammation likely contributes to the pathogenesis of COVID-19, leading to various pathological alterations through the activation of inflammatory cascades, myeloid cell sensing, and inflammasome activation [29] (see Figure 4). Minor alleles of *IL1A* variants were associated with a lower probability of developing radiological affectation. The effect of the genetic variants on IL-1α expression or functioning is unknown. However, our results suggest that *IL1A* variants merit investigation as possible predictors of response to corticoid treatments and their effect on inflammation. Two IFNG Antisense RNA 1 (*IFNG-AS1*) polymorphisms were associated with survival at 90 days. *IFNG-AS1* acts as a positive regulator of interferon-gamma (IFNγ) secretion [64]. IFNγ plays a crucial role in the body’s defence against viruses [35] and is one of the cytokines involved in the cytokine storm [29] (see Figure 4). Our results showed that minor alleles of the *IFNG-AS1* genetic variants were associated with a higher probability of survival at 90 days. Although no previous study investigated *INFG-AS1* polymorphisms in relation to treatment response, the IFNγ levels have been found increased in some patients resistant to corticoids [36]. Our results suggest that *IFNG-AS1* variants may also contribute to response to corticoid treatments in COVID-19 patients.

### 4.3. Genes Related to Response to Corticoids and/or Immunomodulators

Several genes involved in the immune response and corticoid metabolism were found associated with the response in the combined sample of patients treated with corticoids and/or immunomodulators. However, these findings mainly reflect the results obtained in the CORT cohort, suggesting that its larger sample size influenced the outcome of the analyses.

GWAS analyses revealed two *RBFOX1* polymorphisms associated with radiological affectation in the COMB cohort. In our study, minor alleles of *RBFOX1* variants were associated with a lower probability of developing radiological affectation, thus providing evidence for the role of RBFOX1 in corticoid and the immunomodulator response in COVID-19 patients. Additionally, two ATP-binding cassette subfamily G member 1 (*ABCG1*) polymorphisms were associated with radiological affectation in the GWAS study. ABCG1 is responsible for transporting a lipidic across cellular membranes of macrophages and is implicated in regulating cellular lipid homeostasis in other cell types [65]. In our study, minor alleles of *ABCG1* variants were associated with a lower probability of developing radiological affectation. A study on murine models revealed a connection between the deficiency of ABCG1 in alveolar macrophage and pulmonary granulomatous inflammation [66]. Another study suggested that ABCG1 expression is downregulated by TLR4, contributing to inflammation and lipid accumulation in vascular smooth muscle cells, mitigating the PPARγ/LXRα signalling pathway [44] (see Figure 4).

Within the selected candidate genes, several *IFNG-AS1* polymorphisms were associated with survival at 90 days. As explained before, IFNG-AS1 acts as a positive regulator of IFNγ secretion [64], a key component of the immune system [29] (see Figure 4). Minor alleles of the *IFNG-AS1* genetic variants were associated with a higher probability of survival at 90 days. Interestingly, the association was of a higher magnitude than that observed in the CORT cohort, suggesting that there is a relationship between *IFNG-AS1* variants and the response to COVID-19 treatments and to corticoids in particular. However, the association of these *IFNG-AS1* variants with the response to immunomodulators cannot be confirmed due to the limited number of participants treated with this medication. Three *IL-1α* polymorphisms were also associated with radiological affectation in the COMB cohort. Minor alleles of the *IL1A* genetic variants were associated with a lower probability of developing radiological affectation. As explained before, IL-1α participates in the immune response, and it is also related to the cytokine storm [29,34]. It is plausible that, in addition to the corticoid response, IL-1α influences the response to the immunomodulators used to reduce the cytokine storm in COVID-19 patients. The results of this study would support this hypothesis, although no significant association was detected in the subgroup of patients treated with immunomodulators, probably due to its limited sample size. Finally, the rs149895872 variant in the *TLR10* gene was associated with survival at 90 days in the COMB sample. TLR10 has been reported to be the only TLR that exhibits anti-inflammatory properties [43] (see Figure 4). The *TLR10* rs149895872-C allele was associated with an increased mortality risk in treated patients. However, the properties and mechanisms of action of TLR10 are still not clear. Our results suggest that the *TLR10* variant rs149895872 may contribute to the response to corticoid and immunomodulator treatments in COVID-19 patients. However, as in the previous case, the possible association of *TLR10* variants with the response to immunomodulators needs to be investigated in a larger sample.

### 4.4. Pathway Enrichment Analysis

The gene enrichment analyses of the 5000 most significantly associated genes in the GWAs studies revealed enrichment mostly in genes involved in neuronal activity in the biological process and cellular component databases. Previous studies have related COVID-19 infection with neurological inflammation and, consequently, dysregulation of neural cell types [67]. We propose that variants in the neurogenesis can create neurons more susceptible to inflammation. Another study suggested that neurological complications are common in COVID-19 patients [68]. These complications can be considered part of the lack of response of treatments.

However, when analysing molecular functions, overrepresentation of the genes involved in molecular signalling was observed. Interestingly, the most significant cause of severe clinical complications and lack of response to treatment in COVID-19 is the cytokine storm, which is essentially a dysregulation of molecular signalling.

### 4.5. Study Limitations

Our study has several limitations. In addition to the moderate sample size of the IMM cohort, no detailed clinical data were available on the specific symptoms experienced by the patients after hospital discharge, except survival after 90 days. Furthermore, no information on the virus variants present in the recruited patients was available, as the information was not routinely collected during the first and second waves of COVID-19 in Spanish hospitals. It would be interesting to investigate the correlation between the virus characteristics and treatment outcome, which was not possible in our cohorts.

## 5. Conclusions

Many of the genes we found related to COVID-19 treatment response interact with the NF-κB factor (*RBFOX1*, *TLR10*, *TLR2*, *TLR6*, *TLR1*, *TLR5*, *TLR4*, *ABCG1*, *ANK3*, *UGT1A1*, *IFNG-AS1*, and *IL1A*) (see Figure 4). This factor is key in the regulation of the expression of cytokines that produce the cytokine storm and one of the main targets of corticoids. Furthermore, the Immunomodulators used for the treatment of COVID-19 are antibodies that usually target IL-6, IL-6R, and other interleukins that are also components of the cytokine storm. Our results suggest that genetic variants in the pathway of the pro-inflammatory NF-κB factor and related to cytokine storms may constitute predictors of the response to treatments for severe coronavirus infections.

In summary, a number of genetic variants in proteins involved in immune response and cell transport were found associated with the response to corticoids and IMM treatments. Although several different genetic variants were found associated with the response to corticoids and with the response to immunomodulators that may help to select the most adequate treatment, further studies are required to confirm their specificity. If replicated, these findings may help to personalise treatments in future severe coronavirus infections.

## Figures and Tables

**Table 1:** Phenotypic distribution of the study cohorts.

Treatment	Phenotype	*n* (%)
Immunomodulators (IMM)	Survival at 90 days	96 (14) did not survive 580 (86) survived
	ICU	346 (50) ICU 350 (50) No ICU
	Type of Ventilation *	1 = 59 (9); 2 = 286 (41); 3 = 351 (50)
	Radiological affectation	647 (99) affected 9 (1) unaffected
Corticoids (CORT)	Survival at 90 days	315 (17) did not survive 1501 (83) survived
	ICU	449 (23) ICU 1495 (77) No ICU
	Type of Ventilation	1 = 346 (18); 2 = 1034 (53); 3 = 565 (29)
	Radiological affectation	1744 (94) affected 105 (6) unaffected
Immunomodulators and/or Corticoids (COMB)	Survival at 90 days	321 (16) did not survive 1662 (84) survived
	ICU	556 (26) ICU 1621 (74) No ICU
	Type of Ventilation	1 = 346 (16); 2 = 1138 (53); 3 = 669 (31)
	Radiological affectation	1938 (95) affected 110 (5) unaffected

* Type of ventilation (1 = no ventilation; 2 = conventional oxygen therapy; 3 = nasal cannulas and (IMV).

**Table 2:** List of candidate genes related to the primary treatments used for COVID-19.

Treatment related genes	**Type of Drug**	**Drug**	**Main Targets and Metabolic Pathways**
	Corticoids	Dexamethasone	*CYP3A4*, *CYP3A5*, *CYP2B6*, *CYP2C19*, *CYP2C8*, *ABCB1*, *ABCB11*, *ABCC2*
		Hydrocortisone	*CYP3A4*, *CYP3A5*, *CYP11B2*, *CYP2C8*, *CYP1B1*, *CYP2B6*, *CYP2C9*, *CYP2C19*, *ABCB1*
		Prednisone	*CYP3A5*, *CYP2B6*, *CYP2C19*, *CYP2C8*, *ABCB1*, *ABCB11*, *ABCC2*
		Methylprednisolone	*CYP3A4*, *CYP1B1*, *CYP2B6*, *CYP2C8*, *CYP2C19*, *CYP2C9*, *ABCB1*
		Cortisone	*CYP3A4*, *CYP3A5*
	Immuno-modulators	OTHERS	*IL1R1*, *IL1A*, *IL1B*, *IL2*, *IL4*, *IL6*, *IL6R*, *IL10*, *TNFA*, *IFNAR1*
		Tocilizumab	*IL6R*, *CYP3A4*, *FCGR3A*, *UGT1A1*, *FCGR3A*
	Interferon	Interferons	*IFNAR1*, *IFNAR2*, *CYP1A2*, *IFITM3*, *IFNG*, *IFNGR1*, *IFNGR2*, *IFNLR1*, *IFNA16*, *IRF7*
Infection related genes	**Related Function**		**Genes Involved**
	Entry point		*ACE*, *ACE2*, *ABO*
	Cytokine storm		*TLR10*, *TLR8*, *TLR7*, *TLR5*, *TLR4*, *TLR3*, *TLR2*, *TLR1*, *IL1R1*, *IL-1A*, *IL1B*, *IL2*, *IL4*, *IL6*, *IL6R*, *IL10 & TNF-α*, *IFNAR1*, *IFNAR2*, *IFITM3*, *IFNG*, *IFNGR1*, *IFNGR2*, *IFNLR1*, *IFNA16*, *IRF7*

**Table 3:** Summary of the significant findings in the GWAS analyses.

Treatment	Phenotype	Gene	SNP	Allele	OR/	*p*-Value
					BETA	
Immunomodulators cohort (IMM)	Type of Ventilation	*ANK3*	rs144347645_1_	T	−0.64	2.68 × 10^−8^
			rs16915354_1_	T	−0.64	2.68 × 10^−8^
			rs145299149_1_	A	−0.64	2.68 × 10^−8^
			rs142740585_1_	T	−0.64	2.68 × 10^−8^
			rs115701266_1_	A	−0.64	2.68 × 10^−8^
			rs116165734_1_	C	−0.64	2.68 × 10^−8^
			rs16915359_1_	G	−0.64	2.68 × 10^−8^
			rs16915361_1_	C	−0.64	2.68 × 10^−8^
			rs149847098_1_	G	−0.64	4.27 × 10^−8^
			rs144806783_1_	G	−0.64	4.27 × 10^−8^
Corticoids cohort (CORT)	Radiological affectation	*MIR924HG*	rs36036468_1_	T	0.21	4.99 × 10^−8^
		*RBFOX1*	rs551128984_1_	C	0.24	2.01 × 10 ^−8^
		*ZMAT3*	rs74370746_4_	A	0.05	2.33 × 10 ^−8^
			rs78451671_3_	C	0.05	2.33 × 10 ^−8^
Combined cohort (COMB)	Radiological affectation	*RBFOX1*	rs551128984_1_	C	0.23	3.00 × 10^−9^
			rs72765129_1_	G	0.28	4.75 × 10^−8^
		*ABCG1*	rs914110892_2_	A	0.14	1.38 × 10^−8^
			rs112302620_3_	C	0.14	1.38 × 10^−8^

Gene variant locations: intron variant, regulatory region variant, intergenic variant, and upstream gene variant. Beta coefficients are reported for categoric variables (type of ventilation), while odds ratios (ORs) are presented for bimodal variables.

**Table 4:** Summary of the significant findings in the candidate gene studies.

Treatment	Phenotype	Gene	SNP	Allele	OR/Beta	*p*-Value
Immunomodulators cohort (IMM)	Survival at 90 Days	*TLR5*	rs55866312_1_	T	13.91	4.39 × 10^−3^
			rs542741410_1_	T	9.82	5.39 × 10^−3^
	ICU	*ABCB11*	rs3770585_1_	A	1.53	2.55 × 10^−4^
Corticoids cohort (CORT)	Survival at 90 Days	*IFNG-AS1*	rs12306899_1_	C	0.65	4.05 × 10^−5^
			rs12300716_1_	C	0.65	4.05 × 10^−5^
		*TLR1*, *TLR6*	rs111530790_1_	dupT	1.54	2.86 × 10^−4^
			rs6849400_1_	A	1.54	2.86 × 10^−4^
			rs11933455_1_	G	1.55	2.56 × 10^−4^
			rs146468588_1_	T	1.54	3.49 × 10^−4^
			rs376523214_1_	G	1.54	3.49 × 10^−4^
			rs111980996_1_	C	1.54	3.49 × 10^−4^
			rs113668069_1_	G	1.54	3.49 × 10^−4^
			rs148035117_1_	A	1.54	3.49 × 10^−4^
		*TLR10*	rs149895872_2_	C	4.56	9.05 × 10^−4^
	ICU	*CYP2C19*	rs12258243_1_	A	2.65	3.19 × 10^−4^
		*ACE2*	rs62578917_1_	A	3.51	3.26 × 10^−4^
	Type of Ventilation	*TLR4*	rs12377632_1_	C	0.08	3.45 × 10^−4^
			rs7868859_1_	G	−0.09	6.35 × 10^−4^
		*UGT1A1*	rs6742078_1_	T	0.17	5.12 × 10^−4^
	Radiological affectation	*IL-1α*	rs3783585_1_	T	0.07	7.83 × 10^−5^
			rs2071375_1_	T	0.44	5.63 × 10^−4^
			rs697_1_	T	0.44	5.63 × 10^−4^
Combined cohort (COMB)	Survived 90 Days	*IFNG-AS1*	rs12300716_1_	C	0.64	8.18 × 10^−6^
			rs12306899_1_	C	0.65	1.18 × 10^−5^
			rs10878747_1_	A	0.65	1.78 × 10^−5^
			rs10878749_1_	T	0.65	2.51 × 10^−5^
			rs7301797_1_	G	0.66	2.54 × 10^−5^
			rs7306440_1_	G	0.66	2.54 × 10^−5^
			rs2870955_1_	T	0.66	3.60 × 10^−5^
			rs7133171_1_	C	0.66	3.60 × 10^−5^
			rs7137158_1_	C	0.66	3.60 × 10^−5^
			rs11177059_1_	T	0.66	4.74 × 10^−5^
		*TLR10*	rs149895872_1_	C	4.07	1.05 × 10^−3^
	Ventilation	*UGT1A1*	rs6742078_1_	T	0.16	6.73 × 10^−4^
	Radiological affectation	*IL-1α*	rs2071375_1_	T	0.47	1.13 × 10^−3^
			rs697_1_	T	0.47	1.13 × 10^−3^
			rs3783585_1_	T	0.08	6.41 × 10^−5^

Gene variant locations: intronic variant and missense variant. Beta coefficients are reported for the categoric variables (type of ventilation), while odds ratios (OR) are presented for the bimodal variables.

## Data Availability

Summary statistics of the data of the main study [6] have been aggregated with those from the COVID-19 Host Genetics Initiative (https://www.covid19hg.org accessed on 1 April 2022); the results of this study will be shared upon request to the corresponding author.

## References

[1] Molina-Mora J.A., González A., Jiménez-Morgan S., Cordero-Laurent E., Brenes H., Soto-Garita C., Sequeira-Soto J., Duarte-Martínez F. (2022). Clinical Profiles at the Time of Diagnosis of SARS-CoV-2 Infection in Costa Rica During the Pre-Vaccination Period Using a Machine Learning Aroach. Phenomics.

[2] Raghav P.K., Mann Z., Ahluwalia S.K., Rajalingam R. (2023). Potential Treatments of COVID-19: Drug Repurposing and Therapeutic Interventions. J. Pharmacol. Sci..

[3] COVID-19 Vaccinations Have Saved More than 1.4 Million Lives in the WHO European Region, a New Study Finds. https://www.who.int/europe/news-room/16-01-2024-covid-19-vaccinations-have-saved-more-than-1.4-million-lives-in-the-who-european-region--a-new-study-finds.

[4] Noureddine F.Y., Chakkour M., El Roz A., Reda J., Al Sahily R., Assi A., Joma M., Salami H., Hashem S.J., Harb B. (2021). The Emergence of SARS-CoV-2 Variant(s) and Its Impact on the Prevalence of COVID-19 Cases in the Nabatieh Region, Lebanon. Med. Sci..

[5] Gong Z., Song T., Hu M., Che Q., Guo J., Zhang H., Li H., Wang Y., Liu B., Shi N. (2024). Natural and Socio-Environmental Factors in the Transmission of COVID-19: A Comprehensive Analysis of Epidemiology and Mechanisms. BMC Public Health.

[6] Cruz R., Diz-De Almeida S., de Heredia M.L., Quintela I., Ceballos F.C., Pita G., Lorenzo-Salazar J.M., González-Montelongo R., Gago-Domínguez M., Porras M.S. (2022). A Novel Genes and Sex Differences in COVID-19 Severity. Hum. Mol. Genet..

[7] Mo Y., Fisher D. (2016). A Review of Treatment Modalities for Middle East Respiratory Syndrome. J. Antimicrob. Chemother..

[8] Chavda V.P., Vuu S., Mishra T., Kamaraj S., Patel A.B., Sharma N., Chen Z.S. (2022). Recent Review of COVID-19 Management: Diagnosis, Treatment and Vaccination. Pharmacol. Rep..

[9] Hu B., Huang S., Yin L. (2020). The Cytokine Storm and COVID-19. J. Med. Virol..

[10] Valenzuela O., Ibáñez S., Poli M.C., Roessler P., Aylwin M., Roizen G., Iruretagoyena M., Agar V., Donoso J., Fierro M. (2020). First Report of Tocilizumab Use in a Cohort of Latin American Patients Hospitalized for Severe COVID-19 Pneumonia. Front. Med..

[11] Fu B., Xu X., Wei H. (2020). Why Tocilizumab Could Be an Effective Treatment for Severe COVID-19?. J. Transl. Med..

[12] Kotak S., Khatri M., Malik M., Malik M., Hassan W., Amjad A., Malik F., Hassan H., Ahmed J., Zafar M. (2020). Use of Tocilizumab in COVID-19: A Systematic Review and Meta-Analysis of Current Evidence. Cureus.

[13] Biswas M., Sawajan N., Rungrotmongkol T., Sanachai K., Ershadian M., Sukasem C. (2022). Pharmacogenetics and Precision Medicine Aroaches for the Improvement of COVID-19 Therapies. Front. Pharmacol..

[14] Yeh R.F., Gaver V.E., Patterson K.B., Rezk N.L., Baxter-Meheux F., Blake M.J., Eron J.J., Klein C.E., Rublein J.C., Kashuba A.D.M. (2006). Lopinavir/Ritonavir Induces the Hepatic Activity of Cytochrome P450 Enzymes CYP2C9, CYP2C19, and CYP1A2 But Inhibits the Hepatic and Intestinal Activity of CYP3A as Measured by a Phenotyping Drug Cocktail in Healthy Volunteers. JAIDS J. Acquir. Immune Defic. Syndr..

[15] Rendic S., Guengerich F.P. (2020). Metabolism and Interactions of Chloroquine and Hydroxychloroquine with Human Cytochrome P450 Enzymes and Drug Transporters. Curr. Drug Metab..

[16] Tomlinson E.S., Maggs J.L., Park B.K., Back D.J. (1997). Dexamethasone Metabolism in Species Differences. J. Steroid. Biochem. Mol. Biol..

[17] García-Menaya J.M., Cordobés-Durán C., García-Martín E., Agúndez J.A.G. (2019). Pharmacogenetic Factors Affecting Asthma Treatment Response. Potential Implications for Drug Therapy. Front. Pharmacol..

[18] Badary O.A. (2021). Pharmacogenomics and COVID-19: Clinical Implications of Human Genome Interactions with Repurposed Drugs. Pharmacogenomics J..

[19] Fricke-Galindo I., Falfán-Valencia R. (2021). Pharmacogenetics Aroach for the Improvement of COVID-19 Treatment. Viruses.

[20] Pino-Yanes M., Corrales A., Casula M., Blanco J., Muriel A., Espinosa E., García-Bello M., Torres A., Ferrer M., Zavala E. (2010). Common Variants of TLR1 Associate with Organ Dysfunction and Sustained Pro-Inflammatory Responses during Sepsis. PLoS ONE.

[21] San Gil A. (2017). Pneumònia Aguda de La Comunitat En l’Adult: Etiologia i Biomarcadors Genètics de l’Hoste. Ph.D. Thesis.

[22] AL-Eitan L.N., Alahmad S.Z. (2021). Pharmacogenomics of Genetic Polymorphism within the Genes Responsible for SARS-CoV-2 Susceptibility and the Drug-Metabolising Genes Used in Treatment. Rev. Med. Virol..

[23] Franczyk B., Rysz J., Miłoński J., Konecki T., Rysz-Górzyńska M., Gluba-Brzózka A. (2022). Will the Use of Pharmacogenetics Improve Treatment Efficiency in COVID-19?. Pharmaceuticals.

[24] Fatima S., Ratnani I., Husain M., Surani S. (2020). Radiological Findings in Patients with COVID-19. Cureus.

[25] Graffelman J., Moreno V. (2013). The Mid P-Value in Exact Tests for Hardy-Weinberg Equilibrium. Stat. Al. Genet. Mol. Biol..

[26] Taliun D., Harris D.N., Kessler M.D., Carlson J., Szpiech Z.A., Torres R., Taliun S.A.G., Corvelo A., Gogarten S.M., Kang H.M. (2021). Sequencing of 53,831 Diverse Genomes from the NHLBI TOPMed Program. Nature.

[27] AL-Taie A., Büyük A.Ş., Sardas S. (2022). Considerations into Pharmacogenomics of COVID-19 Pharmacotherapy: Hope, Hype and Reality. Pulm. Pharmacol. Ther..

[28] Moreira R.P.P., Jorge A.A.L., Gomes L.G., Kaupert L.C., Filho J.M., Mendonca B.B., Bachega T.A.S.S. (2011). Pharmacogenetics of Glucocorticoid Replacement Could Optimize the Treatment of Congenital Adrenal Hyperplasia Due to 21-Hydroxylase Deficiency. Clinics.

[29] Montazersaheb S., Hosseiniyan Khatibi S.M., Hejazi M.S., Tarhriz V., Farjami A., Ghasemian Sorbeni F., Farahzadi R., Ghasemnejad T. (2022). COVID-19 Infection: An Overview on Cytokine Storm and Related Interventions. Virol. J..

[30] Vohra M., Sharma A.R., Satyamoorthy K., Rai P.S. (2021). Pharmacogenomic Considerations for Repurposing of Dexamethasone as a Potential Drug against SARS-CoV-2 Infection. Per. Med..

[31] Lee J.S., Wang J., Martin M., Germer S., Kenwright A., Benayed R., Spleiss O., Platt A., Pilson R., Hemmings A. (2011). Genetic Variation in UGT1A1 Typical of Gilbert Syndrome Is Associated with Unconjugated Hyperbilirubinemia in Patients Receiving Tocilizumab. Pharmacogenet. Genom..

[32] Fardel O., Payen L., Courtois A., Vernhet L., Lecureur V. (2001). Regulation of Biliary Drug Efflux Pump Expression by Hormones and Xenobiotics. Toxicology.

[33] Jiang Y., Rubin L., Zhou Z., Zhang H., Su Q., Hou S.T., Lazarovici P., Zheng W. (2022). Pharmacological Therapies and Drug Development Targeting SARS-CoV-2 Infection. Cytokine Growth Factor Rev..

[34] Jarczak D., Nierhaus A. (2022). Cytokine Storm—Definition, Causes, and Implications. Int. J. Mol. Sci..

[35] Hasanvand A. (2022). COVID-19 and the Role of Cytokines in This Disease. Inflammopharmacology.

[36] Britt R.D., Thompson M.A., Sasse S., Pabelick C.M., Gerber A.N., Prakash X.Y.S. (2019). Th1 Cytokines TNF-and IFN-Promote Corticosteroid Resistance in Developing Human Airway Smooth Muscle. Am. J. Physiol. Lung. Cell. Mol. Physiol..

[37] Purcell S., Neale B., Todd-Brown K., Thomas L., Ferreira M.A.R., Bender D., Maller J., Sklar P., De Bakker P.I.W., Daly M.J. (2007). PLINK: A Tool Set for Whole-Genome Association and Population-Based Linkage Analyses. Am. J. Hum. Genet..

[38] Takahashi T., Luzum J.A., Nicol M.R., Jacobson P.A. (2020). Pharmacogenomics of COVID-19 Therapies. NPJ Genom. Med..

[39] Zaidat O.O., Erwin Grüter B., Cantonal Hospital A., Luis Rafael Moscote-Salazar S., Yan J., Jiang W., Liu J., Liao X., Zhou J., Li B. (2021). A Rare Variant of ANK3 Is Associated With Intracranial Aneurysm. Front. Neurol..

[40] Yang Y., Zhu Z., Hui L., Sun P. (2024). Effects of CACNA1C and ANK3 on Cognitive Function in Patients with Bipolar Disorder. Prog. Neuropsychopharmacol. Biol. Psychiatry.

[41] Kloth K., Lozic B., Tagoe J., Hoffer M.J.V., Van der Ven A., Thiele H., Altmüller J., Kubisch C., Au P.Y.B., Denecke J. (2021). ANK3 Related Neurodevelopmental Disorders: Expanding the Spectrum of Heterozygous Loss-of-Function Variants. Neurogenetics.

[42] Thoeni C., Waldherr R., Scheuerer J., Schmitteckert S., Roeth R., Niesler B., Cutz E., Flechtenmacher C., Goeert B., Schirmacher P. (2019). Expression Analysis of Atp-Binding Cassette Transporters Abcb11 and Abcb4 in Primary Sclerosing Cholangitis and Variety of Pediatric and Adult Cholestatic and Noncholestatic Liver Diseases. Can. J. Gastroenterol. Hepatol..

[43] Fore F., Indriputri C., Mamutse J., Nugraha J. (2020). TLR10 and Its Unique Anti-Inflammatory Properties and Potential Use as a Target in Therapeutics. Immune Netw..

[44] Cao X., Zhang L., Chen C., Wang Q., Guo L., Ma Q., Deng P., Zhu G., Li B., Pi Y. (2017). The Critical Role of ABCG1 and PPARγ/LXRα Signaling in TLR4 Mediates Inflammatory Responses and Lipid Accumulation in Vascular Smooth Muscle Cells. Cell. Tissue Res..

[45] Bank S., Skytt Andersen P., Burisch J., Pedersen N., Roug S., Galsgaard J., Ydegaard Turino S., Broder Brodersen J., Rashid S., Kaiser Rasmussen B. (2014). Polymorphisms in the Inflammatory Pathway Genes TLR2, TLR4, TLR9, LY96, NFKBIA, NFKB1, TNFA, TNFRSF1A, IL6R, IL10, IL23R, PTPN22, and PPARG Are Associated with Susceptibility of Inflammatory Bowel Disease in a Danish Cohort. PLoS ONE.

[46] Duggan J.M., You D., Cleaver J.O., Larson D.T., Garza R.J., Guzmán Pruneda F.A., Tuvim M.J., Zhang J., Dickey B.F., Evans S.E. (2011). Synergistic Interactions of TLR2/6 and TLR9 Induce a High Level of Resistance to Lung Infection in Mice. J. Immunol..

[47] Chakraborty C., Sharma A.R., Bhattacharya M., Sharma G., Lee S.S., Agoramoorthy G. (2020). Consider TLR5 for New Therapeutic Development against COVID-19. J. Med. Virol..

[48] Kaushik D., Bhandari R., Kuhad A. (2021). TLR4 as a Therapeutic Target for Respiratory and Neurological Complications of SARS-CoV-2. Expert Opin. Ther. Targets.

[49] Gao C., Ren S., Lee J.H., Qiu J., Chapski D.J., Rau C.D., Zhou Y., Abdellatif M., Nakano A., Vondriska T.M. (2016). RBFox1-Mediated RNA Splicing Regulates Cardiac Hypertrophy and Heart Failure. J. Clin. Investig..

[50] Dery K.J., Wong Z., Wei M., Kupiec-Weglinski J.W. (2023). Mechanistic Insights into Alternative Gene Splicing in Oxidative Stress and Tissue Injury. Antioxid. Redox. Signal..

[51] Wang M., Cheng L., Gao Z., Li J., Ding Y., Shi R., Xiang Q., Chen X. (2023). Investigation of the Shared Molecular Mechanisms and Hub Genes between Myocardial Infarction and Depression. Front. Cardiovasc. Med..

[52] Fan J., Long Q.X., Ren J.H., Chen H., Li M.M., Cheng Z., Chen J., Zhou L., Huang A.L. (2022). Genome-Wide Association Study of SARS-CoV-2 Infection in Chinese Population. Eur. J. Clin. Microbiol. Infect. Dis..

[53] Alors-Pérez E., Pedraza-Arevalo S., Blázquez-Encinas R., García-Vioque V., Agraz-Doblas A., Yubero-Serrano E.M., Sánchez-Frías M.E., Serrano-Blanch R., Gálvez-Moreno M.Á., Gracia-Navarro F. (2023). Altered CELF4 Splicing Factor Enhances Pancreatic Neuroendocrine Tumors Aggressiveness Influencing MTOR and Everolimus Response. Mol. Ther. Nucleic Acids.

[54] Engel J.J., van der Made C.I., Keur N., Setiabudiawan T., Röring R.J., Damoraki G., Dijkstra H., Lemmers H., Ioannou S., Poulakou G. (2023). Dexamethasone Attenuates Interferon-Related Cytokine Hyperresponsiveness in COVID-19 Patients. Front. Immunol..

[55] Botton M.R., Whirl-Carrillo M., Del Tredici A.L., Sangkuhl K., Cavallari L.H., Agúndez J.A.G., Duconge J., Lee M.T.M., Woodahl E.L., Claudio-Campos K. (2020). PharmVar GeneFocus: CYP2C19. Clin. Pharmacol. Ther..

[56] Matoulková P., Pávek P., Malý J., Vlček J. (2014). Cytochrome P450 Enzyme Regulation by Glucocorticoids and Consequences in Terms of Drug Interaction. Expert Opin. Drug Metab. Toxicol..

[57] Scott S.A., Sangkuhl K., Shuldiner A.R., Hulot J.S., Thorn C.F., Altman R.B., Klein T.E. (2012). PharmGKB Summary: Very Important Pharmacogene Information for Cytochrome P450, Family 2, Subfamily C, Polypeptide 19. Pharmacogenet. Genom..

[58] Creeden J.F., Gordon D.M., Stec D.E., Hinds T.D. (2021). Bilirubin as a Metabolic Hormone: The Physiological Relevance of Low Levels. Am. J. Physiol. -Endocrinol. Metab..

[59] Gammal R.S., Court M.H., Haidar C.E., Iwuchukwu O.F., Gaur A.H., Alvarellos M., Guillemette C., Lennox J.L., Whirl-Carrillo M., Brummel S.S. (2016). Clinical Pharmacogenetics Implementation Consortium (CPIC) Guideline for UGT1A1 and Atazanavir Prescribing. Clin. Med. Ther..

[60] Yamasaki S., Tanimoto K., Kohno K., Kadowaki M., Takase K., Kondo S., Kubota A., Takeshita M., Okamura S. (2015). UGT1A1 *6 Polymorphism Predicts Outcome in Elderly Patients with Relapsed or Refractory Diffuse Large B-Cell Lymphoma Treated with Carboplatin, Dexamethasone, Etoposide and Irinotecan. Ann. Hematol..

[61] Scialo F., Daniele A., Amato F., Pastore L., Matera M.G., Cazzola M., Castaldo G., Bianco A. (2020). ACE2: The Major Cell Entry Receptor for SARS-CoV-2. Lung.

[62] Finney L.J., Glanville N., Farne H., Aniscenko J., Fenwick P., Kemp S.V., Trujillo-Torralbo M.B., Loo S.L., Calderazzo M.A., Wedzicha J.A. (2021). Inhaled Corticosteroids Downregulate the SARS-CoV-2 Receptor ACE2 in COPD through Suression of Type I Interferon. J. Allergy Clin. Immunol..

[63] O’Beirne S.L., Salit J., Kaner R.J., Crystal R.G., Strulovici-Barel Y. (2021). Up-Regulation of ACE2, the SARS-CoV-2 Receptor, in Asthmatics on Maintenance Inhaled Corticosteroids. Respir. Res..

[64] Stein N., Berhani O., Schmiedel D., Duev-Cohen A., Seidel E., Kol I., Tsukerman P., Hecht M., Reches A., Gamliel M. (2019). IFNG-AS1 Enhances Interferon Gamma Production in Human Natural Killer Cells. iScience.

[65] Oram J.F., Vaughan A.M. (2006). ATP-Binding Cassette Cholesterol Transporters and Cardiovascular Disease. Circ. Res..

[66] McPeek M., Malur A., Tokarz D.A., Lertpiriyapong K., Gowdy K.M., Murray G., Wingard C.J., Fessler M.B., Barna B.P., Thomassen M.J. (2019). Alveolar Macrophage ABCG1 Deficiency Promotes Pulmonary Granulomatous Inflammation. Am. J. Respir. Cell Mol. Biol..

[67] Fernández-Castañeda A., Lu P., Geraghty A.C., Song E., Lee M.H., Wood J., O’Dea M.R., Dutton S., Shamardani K., Nwangwu K. (2022). Mild Respiratory COVID Can Cause Multi-Lineage Neural Cell and Myelin Dysregulation. Cell.

[68] Battaglini D., Lopes-Pacheco M., Castro-Faria-Neto H.C., Pelosi P., Rocco P.R.M. (2022). Laboratory Biomarkers for Diagnosis and Prognosis in COVID-19. Front. Immunol..

